# Roles of rifampicin in drug-drug interactions: underlying molecular mechanisms involving the nuclear pregnane X receptor

**DOI:** 10.1186/1476-0711-5-3

**Published:** 2006-02-15

**Authors:** Jiezhong Chen, Kenneth Raymond

**Affiliations:** 1 School of Pharmacy, Faculty of Science, Technology and Engineering, La Trobe University, Bendigo, Vic 3550, Australia

## Abstract

Rifampicin, an important drug in the treatment of tuberculosis, is used extensively despite its broad effects on drug-drug interactions, creating serious problems. The clinical importance of such interactions includes autoinduction leading to suboptimal or failed treatment. The concomitantly administered effects of rifampicin on other drugs can result in their altered metabolism or transportation that are metabolised by cytochromes P450 or transported by p-glycoprotein in the gastrointestinal tract and liver. This review paper summarises recent findings with emphases on the molecular mechanisms used to explain these broad drug-drug interactions. In general, rifampicin can act on a pattern: rifampicin activates the nuclear pregnane X receptor that in turn affects cytochromes P450, glucuronosyltransferases and p-glycoprotein activities. This pattern of action may explain many of the rifampicin inducing drug-drug interactions. However, effects through other mechanisms have also been reported and these make any explanation of such drug-drug interactions more complex.

## Introduction

Multiple drug therapy is often used with a single patient. For example, in individuals infected with HIV and tuberculosis, both anti-tuberculosis and antiretroviral agents have to be used [1]. During treatment, drug-drug interactions occur when the presence of one drug affects the bioavailability of another through absorption, metabolism or disposition, and therefore affects the treatment and drug toxicity in the patient. The estimated incidence of drug-drug interactions that have a clinical significance ranges from 3% to 20%, depending on how many drugs are taken [2]. These adverse reactions are sometimes life-threatening. Such adverse effects caused by drug-drug interactions could be eliminated by more rational drug use, in which patients receive their medications in appropriate doses [3]. To achieve this use, an understanding of the mechanism of drug-drug interactions is important, so that dosages can then be adjusted correspondingly.

Rifampicin is an effective antibiotic against gram-positive bacteria including mycobacteria, being frequently used currently in the chemotherapy of tuberculosis along with isoniazid, pyrazinamide and ethambutol/streptomycin [4,5]. Most patients can be cured with this, but some present with suboptimal or failed treatment [6,7]. Such failures could, in part, be explained by well known autoinduction leading to decreased drug efficacy whereby the drug stimulates its own metabolism into inactive metabolites and thus loses its effectiveness [8,9]. Suboptimal or failed treatment may also be arise from interactions with other drugs and foodstuffs that affect the metabolism of rifampicin [9-11]. For example, Trikatu, a commonly used herb in Ayurvedic medicine, can significantly reduce the peak concentration of rifampicin probably by slowing stomach emptying and gastrointestinal motility [12]. In addition, rifampicin may also interfere with the metabolism of other concomitantly administered drugs [13]. Thus, rifampicin has been used extensively in clinical studies as a prototypical inducer of drug-metabolizing enzymes and transporters, due not only to autoinduction by itself or induction by other drugs and foodstuffs, but also its broad effects on drug-drug interactions. Many earlier reviews have discussed different aspects of rifampicin metabolism and its effects on the actions of other drugs [13-20]. However; application of molecular biology techniques has led to a rapid accumulation of new knowledge. Nuclear pregnane X receptor (PXR) and constitutive androstane receptor (CAR) have now been discovered and their roles in rifampicin-mediated drug-drug interactions demonstrated [21-28]. Another important protein, p-glycoprotein, has also been shown to interplay with CYP3A4 in rifampicin inducing drug-drug interactions [29]. This paper reviews these recent findings, focusing on the molecular mechanisms of rifampicin-inducing drug metabolism.

### History of rifampicin-inducing drug-drug interactions

Rifampicin-inducing drug-drug interactions were recorded 25 years ago, and those affected include anticoagulants, hypoglycaemia agents and contraceptives [30-32]. The average elimination half-life of hexobarbital was decreased from 624 to 262 min and that of tolbutamide from 292 to 160 min following rifampicin treatment in patients with cirrhosis or cholestasis [31]. Rifampicin at 600 mg/day caused about a 3 fold increase in propranolol's clearance [33]. During concurrent treatment with prednisolone, rifampicin increased the plasma clearance of prednisolone by 45% and reduced areas under curves (AUC) by 66%, and these interactions have been reviewed recently [19,34].

The rifampicin-inducing drug-drug interactions were first discovered by Remmer (1972) to be caused by increased levels of cytochrome P450 (CYP), a large group of heme-containing monoxygenase isoenzymes encoded by a gene superfamily [35-37]. Total human CYP was markedly increased by rifampicin in needle biopsy samples taken from human liver. It was found that concomitant administration of rifampicin and oral contraceptives could lead to failure of the antifertility effect of the latter [38]. Further studies elucidated the interaction to be caused by increased estradiol metabolism [39,40]. Increased metabolism of estrodiol in the presence of rifampicin was caused by the induction of estrogen-2-hydroxylation, which is catalysed mainly by CYP1A2 and also CYP1A1, CYP1B1 and CYP3A4 [41-43]. So far, many different CYPs have been identified. Thus, each individual CYP is assigned to a family, subfamily and number, so CYP1A2 stands for family 1, subfamily A, and isoform 2 [36]. Within the same family, all CYP proteins share at least 40 % sequence identity in their amino acid sequence, and within the same subfamily, at least 55% [37].

In 1998, a member of the nuclear receptor superfamily- PXR was found and named, based on its activation by both natural and synthetic C21 steroids (pregnanes) [15-17]. PXR was related to CYP3A4 and both are highly coexpressed in the liver and intestine. PXR is also activated by CYP3A4 inducers including drugs such as rifampicin, dexamethasone, indinavir, paclitaxel, pesticides such as trans-nonachlor, chlordane, endocrine disruptors such as phthalic acid, nonylphenol and other environmental contaminants such as polychlorinated biphenols [21]. Further experiments have demonstrated that PXR transcriptionally regulates CYP3A4. Thus, PXR can bind to CYP3A4 promoters to activate reporter genes, a process that is disregulated in PXR-null mice [19,44].

**Figure 1:**
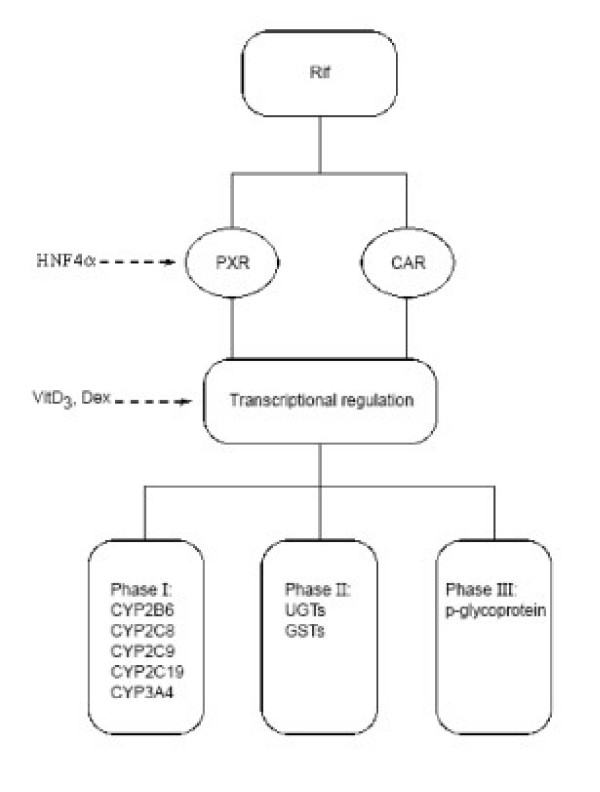
Target genes activated by rifampicin via PXR and CAR. Rifampicin (Rif) induces nuclear receptors PXR and CAR that in turn activate a set of target genes including phase I enzymes such as CYP2B6, CYP2C8, CYP2C9 and CYP3A4, and phase II enzymes such as UGTs, GSTs and phase III enzymes such as p-glycoprotein. PXR and CAR are also regulated by HNF4α and these target genes are also regulated by Vitamin D3, and dexamethasone (Dex).

In addition to CYP3A4, PXR also upregulates a large number of genes involved in xenobiotic detoxification, including enzymes of phase I, II and III drug metabolism (Fig 1). These have been characterised by gene profiling studies performed with pregnenolone 16α-carbonitrile in wild-type and PXR-null mice [45]. The genes regulated by PXR include those encoding CYP enzymes, p-glycoprotein, aldehyde dehydrogenases, UDP-glucuronosyltransferases (UGTs), sulfonyltransferases and glutathione-S-transferases (GSTs).

### Mechanism of induction of rifampicin- role of PXR

**Figure 2:**
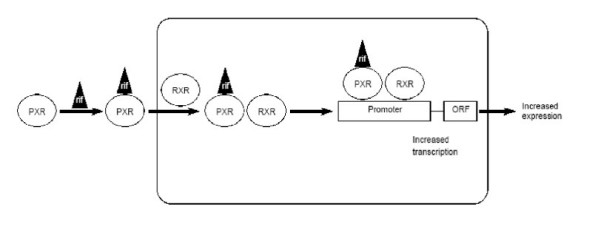
Transcriptional activation of rifampicin target genes. Pregnane X receptor (PXR) binds with rifampicin in the cytoplasm and enters into nucleus to form a heterodimer with retinoic acid receptor (RXR). The heterodimer binds to the promoter of a target gene to activate the transcription of its open reading frame (ORF).

The mechanism of rifampicin-mediated induction of CYP enzymes is now better understood. As mentioned earlier, this induction is mediated by the activation of PXR, which functions as a ligand-activated transcription factor [21,22]. Thus, when a PXR ligand binds to PXR, it in turn activates transcription of CYP 3A4 and several other genes (Fig 2).

The mechanism of PXR in regulating CYP3A4 involves it binding to response elements of the CYP3A4 gene that confers transactivation. PXR binds to CYP3A promoters together with the 9-cis retinoic acid receptor (RXR) as a heterodimer to ER6 (everted repeat with a 6 bp spacer) elements [25,46]. In the CYP3A4 gene, two PXR elements (distant and proximal) have been identified that are separated by more than 7000 nucleotides. Disruption of the distant and proximal elements causes a 73% and 53% decrease in the respective reporter activities. However, *in vitro *binding studies showed that the proximal element was preferred [42]. Chromatin immunoprecipitation experiments showed that the genomic fragment harbouring the proximal element was preferably precipitated over the fragment containing the distant element in the CYP3A4 gene [47]. The fact that CYP3A4 is disregulated in PXR-null mice further demonstrates a role for PXR in CYP3A4 activation [44].

Thus, rifampicin has been clearly identified as a PXR activator by binding to PXR [48]. It also modulates PXR binding to PXR elements of the CYP3A4 promoter [47]. Studies by X-ray crystallography indicated the overall architecture of PXR binding to rifampicin comprised 12 α-helices that fold to form a hydrophobic pocket in the lower portion of the protein [20]. The large volume of the PXR ligand-binding pocket characterised by a smooth, elliptical shape explains why PXR acts as a broad-specificity sensor of lipophilic xenobiotics.

Another receptor, constitutive androstane receptor (CAR), is also involved in CYP3A4 transcriptional regulation. CAR is a member of the steroid/retinoid/thyroid hormone receptor superfamily. It has been demonstrated that CAR can trans-activate CYP3A4 gene expression both *in vitro *and *in vivo *[49]. The CAR response elements are also bound by PXR, indicating their interplay in CYP3A4 expression.

CAR has been demonstrated to cross-talk with PXR, i.e. they share some ligands capable of binding to each other's DNA response elements [44,45,49]. It was also confirmed in chimeric mice with humanized mice liver in which both human CYP3A4 mRNA and protein production was dramatically increased by rifampicin [50]. In receptor-null mice, PXR and CAR regulate overlapping but distinct sets of genes [45]. The significance of cross-talk could be that CAR and PXR together compensate for the loss or malfunction of each other to a certain extent. However, rifampicin has a much lower effect on CAR than PXR [45].

### Effect of rifampicin on CYPs through PXR

CYPs are mainly found on the smooth endoplasmic reticulum membranes in liver hepatocytes and along the intestinal tract mucosal surface [51]. CYPs play an important role in protecting organisms from potentially toxic compounds, by their ability to biotransform lipophilic substrates of diverse structures into more water-soluble metabolites that are subsequently excreted from the body [37,52]. They are also essential for eukaryotic life because of their roles in the metabolism of sterols such as cholesterol, bile acids, fatty acids, prostaglandins, leukotrienes, retinoids as well as biogenic amines [51,53].

These CYPs are important in drug metabolism as they catalyse metabolic reactions involving thousands of substrates in over 60 different types of reactions [54]. Rifampicin can induce many CYPs including CYP2A, CYP2B, CYP2C and CYP3A [51]. Among the many different subsets of these enzymes, CYP3A4 is the most abundant, in both the liver and the intestinal tract. CYP3A4 is responsible for the oxidative metabolism of a wide variety of substrates, involving steroid catabolism and metabolism of foreign compounds, with many of the clinically used drugs being substrates for CYP3A4. It is also a key enzyme in chemical carcinogenesis in both the liver and extrahepatic tissues [55].

The reactions catalysed by CYP3A4 include N-oxidation, C-oxidation, N-dealkylation, O-dealkylation, nitro-reduction, dehydration and C-hydroxylation [55]. CYP3A4 is the primary catalyst of steroid 6β-hydroxylation [56]. These broad reactions are explained by the structure of the CYP3A4 active site which recently has been characterised by X-ray crystallography [57].

CYP3A4 is more efficiently induced than other CYPs [51,58-60]. In primary human hepatocytes, 20 microM rifampicin increased CYP3A4 mRNA by 14 fold, but CYP2B6 by only 2.1 fold [61]. CYP3A4 appears to be regulated transcriptionally by a variety of structurally diverse compounds and hormones [46,62]. In cultured hepatocytes, triiodothyronine decreased CYP3A4 enzyme activity as well as protein and mRNA levels, while dexamethasone and growth hormone increased CYP3A4 gene expression [63].

Rifampicin is involved in drug-drug interaction by its induction of CYPs, especially CYP3A4, and CYP3A4 mRNA is induced by rifampicin in blood mononuclear and liver HepG2 cells [64-66]. The CYP3A4 gene has also been isolated and cloned. A 20-bp region residing -150 bp upstream of the transcription initiation site has been shown to confer responsiveness to rifampicin [67]. The relative potency in inducing CYP3A4 by the rifamycins is rifampicin > rifapentine > rifabutin [10].

Rifampicin can induce both gut (enterocytic) and liver (hepatic) CYP, especially CYP3A4, and increase its own metabolism (autoinduction), but the induced metabolic pathway is still not clear [27]. In healthy volunteers, repeated oral administration of rifabutin (450 mg daily for 10 days) led to lower AUC and minimal blood concentration (Cmin) values, compared to the corresponding theoretical values, with an unchanged half-life [26]. This indicates induced metabolism, although it is unclear as to which specific enzymes are involved. Rifabutin, its derivatives and metabolites, are likely to be metabolised by CYPs, particularly by CYP3A4 [29,68]. If CYPs are involved in rifampicin metabolism, how they act is not clear [29].

There are marked individual variations in the expression of CYP that affect the biological response (efficacy and/or toxicity) of many drugs as well as individual responses to CYP inducers [62,69]. In humans, a 10-fold variation exists among individuals for rifampicin inducing CYP3A4 expression [70,71]. In the liver, substantial interindividual differences in CYP3A4 expression have been detected that could not be explained by environmental factors [70]. A similar situation was found in the intestine whereby CYP3A4 mRNA levels in biopsies of mucosa of five healthy subjects varied by about 10 fold [71]. These differences could in part account for interpatient differences in the oral bioavailability of CYP3A4 substrates.

It has been well demonstrated that rifampicin-induced CYP2C9 is mediated by PXR [72]. Deletion and mutagenesis studies showed that the functional PXR-responsive element is located between -1839/-1824 base pairs upstream from the translation start site previously identified to be CAR-responsive element, and essential for rifampicin inducibility. A second CAR- responsive element located at -2899/-2883 also has a cooperative effect.

In addition, PXR and CAR activate CYP2B6 by transcriptional regulation, although not all CYP2B6 inducers activate PXR [73]. A phenobarbital-responsive enhancer module (PBREM) has been identified, although for human CYP2B6, PBREM effects only a weak response to PXR and CAR. A novel nuclear receptor response module that strongly binds to both PXR and CAR has been described and is known as the xenobiotic-responsive enhancer module (XRE) [74].

### Effect of rifampicin on glucuronosyltransferases through PXR

Rifampicin can induce UDP-glucuronosyltransferase 1A (UGT1A) that catalyses the glucuronidation of a wide range of xenobiotics and endogenous substrates [48,75,76]. UGT1A is regulated by many factors in a tissue-specific manner [77,78]. Both PXR and CAR mediate expression of UGT1A [79-81]. Transgenic mice expressing a constitutively active form of human PXR show a markedly increase of UGT activity [79]. A XRE in the UGT1A gene was thought to be responsible for this induction [80]. The rifampicin mediated-UGT increase may lead to increased clearance of steroids, heme, environmental toxins and drugs [79].

### Effect of rifampicin on p-glycoprotein through PXR

Studies in humans have long indicated that drug transporters are involved in rifampicin hepatic and intestinal uptake. p-Glycoprotein is a plasma membrane-bound drug efflux (multidrug resistance pump), a 170 kDa phosphorylated and glycosylated protein, 1280 amino acids long, which consists of two homologous halves of 610 amino acids joined by a flexible 60 amino acid linker [82]. p-Glycoprotein belongs to the ATP binding cassette superfamily of transport proteins encoded by the multidrug resistance genes (MDR) and was found primarily in drug-eliminating organs, including the kidney, liver, brain, intestinal lumen, testes, adrenal gland, pregnant uterus, tumour cells and epithelial cells [83,84]. They play an important role in drug absorption and disposition, as a biological barrier by extruding toxins and xenobitics from cells rather than by enhancing the excretion of drugs from hepatocytes and renal tubules. Human p-glycoprotein plays an important role in drug efflux through these barriers. Recent paired human tissue studies have shown p-glycoprotein and CYP3A4 protein contents in intestine were higher than in liver, which might indicate an important role for the intestine in drug metabolisms [85].

Should the p-glycoprotein be induced by rifampicin, for example, then the drug's therapeutic usefulness will decline as more drugs are pumped out of the cells. p-Glycoprotein has only recently been identified and thus the potential risk of p-glycoprotein-mediated drug interactions has probably been underestimated in the past. Many drug interactions have been shown to involve both p-glycoprotein and CYP3A4 due to overlapping substrate specificity between them, and similarities in their inhibitors and inducers, as for example, with the coinduction of CYP3A4 and p-glycoprotein by rifampin in human LS180 colon carcinoma cells [86]. Furthermore, p-glycoprotein can control drug access to intracellular CYP3A4 through repeated cycles of drug absorption and efflux, as demonstrated by the studies with cyclosporine, a p-glycoprotein and CYP3A4 inhibitor and GG918, a p-glycoprotein inhibitor only [23]. After inhibition of GG918, K77, a substrate for p-glycoprotein and CYP3A4, accumulated in the cell and metabolism was increased, but felodipine, a substrate of CYP3A4 but not p-glycoprotein was not changed [23]. Cyclosporine decreased the metabolisms of both K77 and felodipine. In another study, inhibition of p-glycoprotein increased both intestinal uptake and blood concentrations of verapamil but with no changes in its rate of metabolism [87].

Rifampicin both induces p-glycoprotein expression and reduces digoxin concentrations during concomitant therapy [88,89]. In healthy volunteers, oral administration of digoxin with rifampicin showed lower plasma concentrations than in those without. This was less pronounced if digoxin was given intravenously. Correspondingly, p-glycoprotein content was 3.5 fold higher after rifampicin treatment [88]. Any regulation is likely to take place at the transcriptional level since increased mRNA levels were detected [90-92]. In healthy subjects, administration of 600mg rifampicin for 9 days upregulated p-glycoprotein mRNA synthesis [92].

The induction of MDR1, a gene encoding p-glycoprotein, is also activated by PXR which binds to PXR response elements located upstream of the MDR1 gene [93]. Only one DR4 (direct repeat with a 4 bp spacer) motif (at about -8 kilobase pairs) was shown to be necessary for the induction by rifampicin, although several other binding sites were identified. It has been demonstrated in mouse hepatoma cell culture and rats that dexamethasone induces the expression of MDR1 and MDR3, with two genes encoding for p-glycoprotein in the mouse [94,95].

### Other factors

PXR is also regulated by a hepatic nuclear factor 4α(HNF4α), which regulates many genes preferentially expressed in liver [22]. HNF4α is stimulated by hepatic maturation factors, such as oncostatin and matrigel, and plays an important role in early embryogenesis [96]. In a fetal hepatocyte primary culture system, inactivation of HNF4α by infection with an adenovirus containing the Cre gene leads to suppression of PXR [97]. An HNF4α antisense RNA has been transducted into human hepatocytes, which resulted in the reduction of HNF4α protein and CYP3A4 [98].

An HNF4α binding site has been characterised in the PXR promoter that is necessary for expression of PXR in fetal hepatocytes [97]. Thus, HNF4α is identified as the key transcriptional factor for PXR. A specific cis-acting element of gene enhancer in CYP3A4 has also identified which confers HNF4α binding and permits PXR- and CAR-mediated gene activation [22].

The molecular mechanisms that underlie transcriptional activation of CYP3A4 are complex involving regulatory factors other than PXR and CAR. These include glucocorticoid and Vitamin D receptors. The CYP3A4 promoter may also be regulated through mechanisms other than PXR effects, including glucocorticoid-induced CYP3A4 gene expression, through the established PXR-dependent pathway and a PXR-independent pathway [99]. Disruption of a responsive element in the CYP3A4 proximal promoter for hepatocyte nuclear factor 3 and CCATT-enhancer binding protein alpha affects its induction by glucocorticoids, dexamethasone and hydrocortisone, but not that by rifampicin. Vitamin D3 has also been shown to induce CYP3A4 independently of PXR [100-102].

### Explanation of drug-drug interactions

Some of the drugs affected by rifampicin are listed in Table 1. Theoretically, any drugs or foods that affect the activation or inhibition of PXR, and/or CYP3A4 and/or p-glycoprotein will interact. CYP3A4 catalyse the metabolism of more than 60% of all drugs currently in use including contraceptive steroids, immunosuppressive agents, imidazole antimycotics and macrolide antibiotics. "Foods" such as grapefruit juice, black pepper inhibit CYP3A4 so causing increased drug levels with resulting therapeutic complications [89,103,104].

**Table 1:** Example drugs affected by rifampicin through different mechanisms

Mediated proteins		p-glycoprotein	CYP1A2	CYP2B6	CYP2C9	CYP2C19	CYP3A4
Affected drugs	Anesthetics:		Ropivacaine [118]			Cyclophosphamine [74]	Alfentanyl [13] Ropivacaine [118]
	Analgesics:		Mexiletine [119]				Codeine [19] Dapsone [19]
	Anti-arrhythmics:						Mexiletine [119] Propafenone [120] Quinidine [19]
	Anti-cancer drugs:		Cyclophosphamide [74] Ifosfamide [74]	Cyclophosphamide [74] Ifosfamide [74]	Cyclophosphamide [74]		Cyclophosphamide [74] Imatinib [121] Tamoxifen [19]
	Anti-epileptics:				Mephenytoin [122]		
	Anti-histamines:	Fexofenadine [123] Terfenadine [123]					Fexofenadine [123] Terfenidine [123]
	Anti-microbials:	Erythromycin [124]					Clarithromtcin [13] Erythromycin [124] Itraconazole [13] Ketoconazole [13] Miconazole [13]
	Benzodiazepines:						Alprazolam [13] Diazepam [13] Midazolam [13] Triazolam [13]
	Cardiac drugs:	Digoxin* [88] Quinidine [13]	Propranolol [13] Verapamil [19]	Bupropion* [114] Methadone [19]	Propranolol [13] Warfarin* [113]	Propranolol [12]	Nifedipine [13] Propranolol [13] Verapamil [19]
	CNS drugs:		Amitriptyline [125] Bupropion [114] Clomipramine [125] Clozapine [19]		Amitriptyline [125] Clomipramine [125]	Amitriptyline [119] Clomipramine [119] Phenytoin [120] Clozapine [19]	Amitriptyline [125] Buspirone [19] Clomipramine [125] Zolpidem [19]
	Gastro-enterology drugs:		Ondansetron [19]				
	HIV Antivirals:	Indinavir [13] Nelfinavir [13] Ritonavir [13]		Efavirenz [13]	Nelfinavir [13]	Nelfinavir [12]	Amprenavir [13] Delavirdine [13] Indinavir [13] Nelfinavir [13] Nevirapine [13] Ritonavir [13] Saquinavir [13]
	HMG CoA Reductase Inhibitors:				Fluvastatin [109] Losartan [109]	Cerivastatin [103]	Atorvastatin [109] Cerivastatin [109] Lovastatin [109] Simvastatin * [108]
	Hypoglycemics			Pioglitazone [127]	Glibenclamide [13] Glipizide [13] Nateglinide [13] Rosiglitazone* [110] Tolbutamine [13]	Repaglinide [12]	Pioglitazone [127]
	Immune Modulators:	Cyclosporine [105] Tacrolimus [17] Methotrexate [19]					Cyclosporine* [105] Sirolimus [17] Tacrolimus [19]
	Steroids:	Cortisol [19] Corticosterone [19] Dexamethason [19]	Estradiol [19]				Estradiol [19] Ethinyloestradiol [19] Hydrocortisone [19]

The drugs affected by or those that affect rifampicin have been extensively reviewed elsewhere [6-11,13,14]. However, the mechanisms for these interactions are not well studied, especially now that considerable progress has been made in understanding the molecular mechanisms of rifampicin. The following examples are selected to represent the major clinically significant rifampicin-inducing drug-drug interactions mainly via the target genes CYP3A4, CYP2C9, CYP2B6 and p-glycoprotein, and these are labelled with * in Table 1.

#### Cyclosporine (CYP3A4)

Interaction of the immunosuppressant cyclosporine and rifampicin is well documented. Doses of cyclosporine had to be increased about 3 fold to maintain its therapeutic blood concentration when coadministrated with rifampicin [105]. A case report has shown that intravenous rifampicin injection led to failure of bone marrow transplantation resulting from subtherapeutic cyclosporine blood levels [106].

Cyclosporine is mainly metabolized by CYP3A4 in human liver [107]. Dexamethasone-induction of cyclosporine metabolism is inhibited by anti-CYP3A4 antibodies *in vitro *and so it is quite likely that rifampicin-inducing cyclosporine metabolism is mediated via CYP3A4 although this has not yet been confirmed by *in vitro *experiments [71].

#### Simvastatin (CYP3A4)

Simvastatin is used to treat hypercholesterolemia and is metabolised to inactive metabolites by CYP3A4. Rifampicin has been shown to reduce its effective concentration while diltiazem, a CYP3A4 inhibitor increases its concentration [108]. In healthy volunteers, 5 day pre-treatment with rifampicin at a dose of 600 mg daily following a single 40 mg dose of simvastatin led to reduced plasmid concentrations of simvastatin of 87 % compared with the control [109]. Thus, concomitant administration of rifampicin and simvastatin can lead to greatly reduced cholesterol lowering efficiency of simvastatin.

#### Rosiglitazone (CYP2C9)

Rosiglitazone is a novel thiazolidinedione antidiabetic drug. Its interaction with rifampicin has been studied in a clinical trial [110]. When ten healthy subjects were administered 8 mg of rosiglitazone after a 6 day pre-treatment of rifampicin at a dose of 600 mg daily, rifampicin decreased the plasma concentration of rosiglitazone by 65% and its elimination half-life from 3.9 to 1.5 hrs. Because rosiglitazone is metabolised by CYP2C8 and CYP2C9, it is proposed that the induction of these CYPs is the basis for this interaction [110,111].

#### Warfarin (CYP2C9)

Warfarin is an anticoagulant drug metabolized by CYP2C9 to 7-hydroxywarfarin [72,112]. Rifampicin increases the rate of clearance of warfarin, thus reducing its effect. In a patient receiving concomitant treatment with rifamycin and warfarin, a 233% increase in warfarin dosage over 4 months was insufficient to attain a therapeutic level and more aggressive upward titration of dose was needed [14,13]. This may be the result of rifampicin induced CYP2C9, which in turn accelerated warfarin metabolism.

#### Bupropion (CYP2B6)

Bupropion, an antidepressant drug, is metabolized by CYP2B6. Bupropion is hydroxylased by microsomes containing cDNA-expressed CYP2B6 and the reaction is subject to 95% inhibition by CYP2B6 inhibitory antibody [114]. High interdividual variability is thought to be a consequence of interactions between environmental and genetic influences on CYP2B6 gene function [115]. Thus, rifampicin induction of CYP2B6 should lead to increased bupropion metabolism, but no data are available to support this view.

#### Digoxin (p-glycoprotein)

Clinical observations have shown that digoxin serum concentration decreased considerably in patients who were treated with rifampicin. This is of special interest since digoxin is generally considered to be renally eliminated [116,117]. In healthy volunteers, co-administration of digoxin and rifampicin orally resulted in plasma concentration of digoxin being much lower during rifampicin treatment. However, the effect was less pronounced after intravenous administration of digoxin [88].

The interaction is mainly mediated by p-glycoprotein in the intestine and intestinal p-glycoprotein content increased 3.5 fold on treatment with rifampicin, which correlated with the decrease in its plasma concentration after oral administration of digoxin [88]. Thus, induction of p-glycoprotein could explain the rifampicin-digoxin interaction.

### Rifampicin-inducing hepatotoxicity

Rifampicin is very toxic to liver, this having been found both in the treatment of tuberculosis and cholestasis [128-130]. When it was used together with pyrazinamide, a 5.8% incidence of severe liver injury was reported, 2.6% with isoniazid and 1.1% alone [130]. The pathogenesis of hepatotoxicity is poorly understood although limited evidence has been obtained. Histopathological examination showed dose-related hepatic necrosis, ballooning degeneration and inflammatory infiltratrates [130,131]. A possible explanation is the increased CYP related effects on calcium homeostasis [132]. It may also be that via oxidant stress, increased lipid peroxidation occurs [133]. In the case when rifampicin and isoniazid are used together, rifampicin may increase isoniazid toxicity though induction of CYPs because acetyl-isoniazid from isoniazid is converted to monoacetyl hydrazine, which is catalysed by CYPs to other hepato-toxic compounds. There were no reports if nuclear receptors were involved in this pathogenesis.

## Conclusion

A common pattern for the molecular mechanisms of rifampicin-inducing drug-drug interaction is rifampicin – PXR/CAR – CYPs, UGTs, GSTs and p-glycoprotein. The possible pathways for rifampicin effects on other drugs are summarised in Table 1. However, other drugs may act in a similar way to rifampicin, or by other pattern as seen with dexamethasone. Therefore, how they act individually is probably complex.

More studies are needed to further increase our knowledge of rifampicin induced drug-drug interactions. Firstly, aspects of the molecular mechanism including how rifampicin activates PXR and modulates their binding to the promoters of CYP3A4 and other target genes should be explored. Secondly, the characterisation of drugs affected by rifampicin and elucidation of their mechanisms of interactions are urgently needed.

## List of abbreviations

AUC: areas under curves

CAR: constitutive androstane receptor

CYP: cytochrome P450

DR4: direct repeat with a 4 bp spacer

ER6: everted repeat with a 6 bp spacer

HNF 4α: hepatic nuclear factor 4α

GSTs: glutathione-S-transferases

MDR: multidrug resistance genes

PBREM: phenobarbital-responsive enhancer

PXR: pregnane X receptor

RXR: 9-cis retinoic acid receptor

UGTs: UDP-glucuronosyltransferases

UGT1A: UDP-glucuronosyltransferase 1A

XRE: xenobiotic response element

## Competing interests

The author(s) declare that they have no competing interests.

## Authors' contributions

KR and JC discussed the most recent progress in the field and formed the concept for the review. JC did the literature search and prepared the first draft of the manuscript. KR then revised and approved this version of the paper to be submitted for publication.
